# Pre- and Postintervention Factor Structure of Functional Independence Measure in Patients with Spinal Cord Injury

**DOI:** 10.1155/2017/6938718

**Published:** 2017-12-21

**Authors:** Mikhail Saltychev, Janne Lähdesmäki, Petteri Jokinen, Katri Laimi

**Affiliations:** ^1^Department of Physical and Rehabilitation Medicine, Turku University Hospital and University of Turku, Turku, Finland; ^2^Division of Clinical Neurosciences, Turku University Hospital and University of Turku, Turku, Finland

## Abstract

**Objective:**

To evaluate the factor structure of Functional Independence Measure (FIM®) scale amongst people with spinal cord injury (SCI).

**Methods:**

This was a retrospective, register-based cohort study on 155 rehabilitants with SCI. FIM was assessed at the beginning and at the end of multidisciplinary inpatient rehabilitation. The internal consistency of the FIM was assessed with Cronbach's alpha and exploratory factor analysis was employed to approximate the construct structure of FIM.

**Results:**

The internal consistency demonstrated high Cronbach's alpha of 0.95 to 0.96. For both pre- and postintervention assessments, the exploratory factor analysis resulted in 3-factor structures. Except for two items (“walking or using a wheelchair” and “expression”), the structures of the identified three factors remained the same from the beginning to the end of rehabilitation. The loadings of all items were sufficient, exceeding 0.3. Both pre- and postintervention chi-square tests showed significant *p* values < 0.0001. The “motor” domain was divided into two factors with this 2-factor structure enduring through the intervention period.

**Conclusions:**

Amongst rehabilitants with SCI, FIM failed to demonstrate unidimensionality. Instead, it showed a 3-factor structure that fluctuated only little depending on the timing of measurement. Additionally, when measured separately, also motor score was 2-dimensional, not 1-dimensional. Using a total or subscale FIM, scores seem to be unjustified in the studied population.

## 1. Introduction

The Functional Independence Measure (FIM) was developed in the 1980s–1990s as a scale measuring a need for assistance in the medical system in the United States [1]. Since then, FIM has been extensively used around the world for diverse health disorders [2–10].

The best known FIM outcome is a radar diagram showing the distribution of individual scores obtained from 18 items of FIM [11]. In addition to this graphical output, total scores and scores for two subscales (“motor” an “cognitive”) are used to describe the severity of disability and changes in functioning over time. The usefulness of these scores has been questioned [5, 12–17]. To be reliable, a score should represent a unified outcome on an interval scale. This means that a scale must represent only one underlying construct, that is, pain or disability severity, to be able to produce a reliable numeric score of several items. This unidimensionality of FIM has not been established yet. Instead, numerous studies have reported that FIM is a multidimensional scale and, because of this multidimensionality, aggregated numeric scores may be meaningless [5, 12–17]. Such studies have been conducted amongst stroke survivors, elderly patients, and SCI patients amongst others. While the multidimensionality of FIM has become well known, there is no knowledge if its factor structure prevails the same over time. To make a comparison between reliable repeated measurements, a scale must preserve its factor structure over time. In other words, the factor structures of FIM should be similar regardless of the timing of measurement, at the beginning or at the end of intervention or even years after. This kind of structure stability of FIM has not been studied so far.

Even if the FIM has been widely used amongst people with SCI [3, 4, 8, 9, 18–25], previous evidence on FIM validity in SCI is scarce. A few studies have reported that FIM may have a substantial ceiling effect in the cognitive subscale and a possible item redundancy within the motor subscale [20, 21] with poor psychometric properties assessed with the Rasch analysis [8]. Based on the Rasch analysis, the FIM motor score was not recommended for use, not at least as a raw sum of item scores. The cognitive scale was found to work well except for the substantial ceiling effect [8]. Due to the uncertainty concerning the reliability of FIM amongst people with SCI, other scales have been suggested to replace FIM in that population, such as Spinal Cord Independence Measure (SCIM) [26].

The objective of this study was to evaluate the factor structure of FIM amongst SCI rehabilitants.

## 2. Methods

This was a retrospective, register-based cohort study amongst 155 patients with SCI who participated in a multidisciplinary inpatient rehabilitation in a university hospital clinic between January 2010 and April 2017. The FIM was filled out by nurses certified to use FIM except for walking at stairs, which was assessed by a trained physiotherapist. The first assessment was usually performed within two days after the admission. In case of a register-based study, no approval of an ethical committee was needed.

In Finland, the rehabilitation of stroke is arranged in several different standardized phases. Subacute rehabilitation is separated from the acute treatment and rehabilitation of SCI. Thus, in this study on subacute rehabilitation, the patients were admitted for multidisciplinary rehabilitation usually after a few months after onset. The main goal of rehabilitation team (a physician, a nurse, a physiotherapist, an occupational therapist, a psychologist, and a social worker) at that subacute stage is to map the situation and prepare the return of a patient to his or her community—even if it is going to happen much later after next several months. After relatively short (a few weeks) stay at a hospital rehabilitation ward, patients with SCI are transferred to one of the five SCI centers for a longer rehabilitation that usually takes a few months. The rehabilitation ward also arranges rehabilitation for patients with SCI who already have returned to their homes.

The FIM has been broadly described previously [11]. The FIM consists of 18 items. Each item is valued on a Likert-like scale from one (“total assistance in all areas”) to seven (“completely independent”). Three sum scores are calculated and documented in a patient's record: a “motor” score (the sum of the scores collected from the first 13 items), a “cognitive” score (the sum of the scores collected from the last five items), and a total score, that is, the sum of motor and cognitive scores. The maximum total score is 126 points, while the minimum is 18 points. The FIM items can be seen in Tables 1 and 2.

### 2.1. Statistical Analysis

The internal consistency of the FIM was assessed with Cronbach's alpha along with its one-sided 95% confidence limit (95% CI). Alpha ≥ 0.9 was considered excellent, ≥0.8 good, ≥0.7 acceptable, ≥0.6 questionable, ≥0.5 poor, and <0.5 was considered unacceptable. This study employed exploratory factor analysis to approximate the construct structure of FIM. The goal was to determine if FIM measures only one latent trait (e.g., disability or need for assistance) or if there are other possible significant latent variables affecting the results as well. The results were analyzed both numerically and graphically. Exploratory factor analysis (principal factors) was applied with a minimum eigenvalue for retention set at >1.0 (Kaiser's rule). The results of factor analysis were rotated by an orthogonal Varimax rotation, assuming that there is no correlation between factors. Retained and excluded factors were also explored visually on a scree plot including a parallel analysis. The demographic characteristics were reported as percentages when appropriate. Otherwise, means, ranges, standard deviations (SDs), and interquartile ranges (IQRs) were reported. All the analyses were performed using Stata/IC Statistical Software, Release 14, StataCorp LP (College Station, TX, USA).

## 3. Results

The median age of the 155 patients was 61 (IQR: 50 to 69) years (Table 3). Of them, 87 (56%) were men and 68 (44%) were women (Table 2). Of these patients, 63 (41%) had paraplegia, 67 had tetraplegia (43%), and 25 (16%) could not be exactly specified by the retrospective data. The multidisciplinary rehabilitation program started (median) 3.1 (IQR: 1.2 to 13.1) months after the SCI onset. The median duration of rehabilitation was 17 (IQR: 11 to 32) days. Therapy and training were started at the day of admission. FIM measurements were conducted (median) within 2 days (IQR: 1 to 3) after admission and within 1 day (IQR: 0 to 2) before the day of discharge from the rehabilitation ward. The median FIM total scores were 107 points at the beginning and 114 points at the end of rehabilitation.

The internal consistency demonstrated high Cronbach's alpha of 0.95 (95% CI: 0.94) before and 0.96 (95% CI: 0.95) after the intervention.

**Figure 1:**
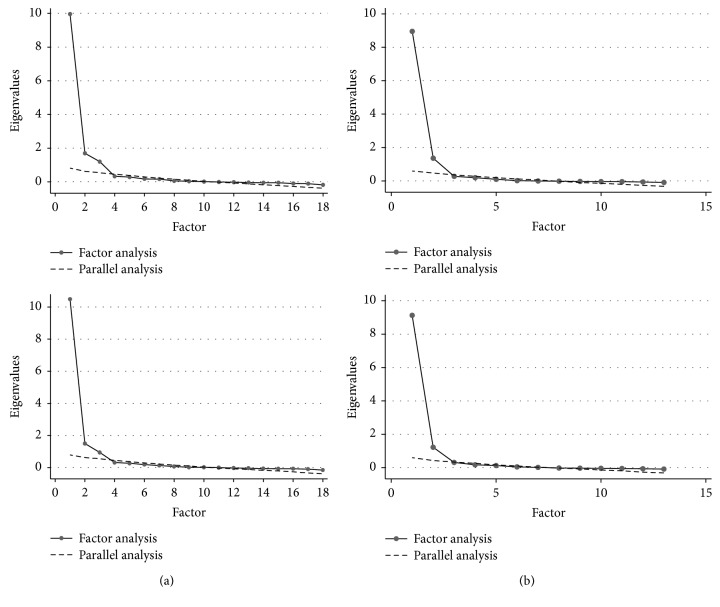
(a) Scree plots of exploratory factor analysis of FIM total score at the beginning and at the end of the rehabilitation course. (b) Scree plots of exploratory factor analysis of FIM motor score at the beginning and at the end of the rehabilitation course.

For both pre- and postintervention assessments, the exploratory factor analysis resulted in 3-factor structures (Figure 1 and Table 1). The eigenvalues for these three factors were 10.0, 1.7, and 1.2 when assessed at the beginning and 10.5, 1.5, and 0.9 at the end of rehabilitation. Even if the third factor had a posttest eigenvalue below Kaiser's cutoff of 1.0, the parallel analysis of scree plot was considered to produce a more precise and logically acceptable result.

Except for two items (“walking or using a wheelchair” and “expression”), the structures of the identified three factors remained the same at the beginning and at the end of rehabilitation. The item “walking or using a wheelchair” migrated from factor 2 to factor 1, and the item “expression” from factor 3 to factor 2. The loadings of all the items were sufficient, exceeding 0.3 [27]. Both pre- and postintervention chi-square tests showed significant *p* values < 0.0001.

The separate factor analysis of only motor score revealed a 2-factor structure in both pre- and posttesting. These two factors had eigenvalues of 8.96 and 1.36 in pretest analysis and 9.13 and 1.22 in posttest analysis, respectively. First factor with much larger eigenvalue was connected to bathing, dressing lower body, toileting, sphincter control, transfers, and using stairs. In turn, second factor was related to eating, grooming, dressing upper body, and moving by walking or moving using a wheelchair. These associations prevailed from pre- to posttesting.

## 4. Discussion

In this retrospective register-based study amongst SCI patients who participated in multidisciplinary inpatient rehabilitation, four main results were observed. First, FIM scale was not unidimensional. Instead, it demonstrated a 3-factor structure. Second, this 3-factor structure was observed by both repeated measurements, at the beginning and at the end of rehabilitation. Third, there were small fluctuations in the intrinsic constructions of these factors: the loadings of some items varied at different time points. Fourth, while so-called “cognitive” subscale of FIM formed (as could be expected) one relatively stable factor, “motor” subscale unexpectedly disintegrated into two distinct factors at both pre- and postintervention time points.

Even if the results were statistically significant, the sample of 155 patients may be too small to examine an 18-item scale and to represent all people with SCI in general. The time interval of two or three weeks between the repeated measures was probably too short to detect small changes in FIM scores or the FIM factor structure. This limitation might also affect the psychometric properties of FIM as the assessors, when scoring the final situation, almost certainly still recalled the initial scores resulting in reduced test-retest reliability.

The multidimensional nature of FIM observed in this study was not a surprise, as previous studies have expressed their concern about the multidimensionality of FIM and, as a result, the uncertainty in calculating the FIM total score [5, 12–17]. Indeed, the total score that measures more than one construct is not as important as was originally thought. We, however, expected at least to see the 2-dimensionality familiar to all FIM users: “motor” and “cognitive” domains. These two dimensions were not confirmed. Instead, three different factors were observed. This kind of multidimensionality has been reported before in elderly people with multiple causes of disability [28], but this phenomenon has not been described earlier amongst people with SCI.

Except for the item “expression” (which migrated between two factors during the intervention), the cognitive domain remained relatively stable including, as expected, last five items of FIM. Instead, the “motor” domain was divided into two factors. The motor domain's 2-factor structure endured through the intervention period with small fluctuations. When reporting problems with factor structure of FIM total score, previous studies usually assumed that, when evaluating separately, motor and cognitive scales should demonstrate unidimensionality. That was not the case in the present study when motor scale was apportioned into two factors. This finding may signal that caution should be applied not only when dealing with the FIM total score but also when interpreting its motor score separately.

The results of the present study yield unambiguous and clinically significant suggestions. As a unidimensional scale, the cognitive subscale of FIM seemed to be able to measure reliably the need for assistance in cognitive functioning. However, the cognitive subscale of FIM could be used in SCI patients with some reservations, as it has to be taken into account that its intrinsic factor structure may fluctuate depending on the timing of measurement. The results are questioning the use of a FIM total or motor score within the studied population as both constructs were found to be multidimensional and, therefore, they may be unable to demonstrate an outcome measured on a unified interval scale.

These considerations do not mean that FIM should not be used at all. FIM has been widely used for 30 years, and the scale holds the status of a worldwide standard assessment of disability (or to be more precise, the assessment of a need for assistance). Such scales are rare and are of a great value as they produce the results comparable across different settings, disorders, and cultures. Our results suggest, however, that (in the studied population of rehabilitants with SCI) a total score or subscores have only limited values. Instead, we would recommend using individual scores obtained from each of 18 items of FIM assessment and report these results as a radial diagram familiar to all FIM users. We also suggest employing informal descriptions in patients' reports to define the restrictions more widely.

Further research conducted on larger samples in different settings and with a longer time interval between repeated measurements may amplify our findings. Considering the widespread use of FIM, the need for such research is apparent. The validity of FIM scores is important not only to health professionals but also to stakeholders when weighing the effectiveness and efficiency of a particular intervention based on the improvements measured by FIM scores.

## 5. Conclusions

Amongst rehabilitants with SCI, FIM failed to demonstrate unidimensionality. Instead, it showed a 3-factor structure that fluctuated only little depending on the timing of measurement. Additionally, when measured separately, also motor score was 2-dimensional, not 1-dimensional. Using a total or subscale FIM, scores seem to be unjustified in the studied population.

## Figures and Tables

**Table 1:** Exploratory factor analysis of FIM total score at the beginning and at the end of rehabilitation (Varimax rotated loadings).

FIM items	Pretest				Posttest			
	Factor 1	Factor 2	Factor 3	Uniqueness	Factor 1	Factor 2	Factor 3	Uniqueness
Self-care								
(A) Eating	0.20	0.80	0.12	0.30	0.22	0.81	0.23	0.24
(B) Grooming	0.39	0.81	0.22	0.14	0.42	0.82	0.19	0.11
(C) Bathing	0.79	0.46	0.08	0.15	0.80	0.43	0.15	0.16
(D) Dressing, upper body	0.46	0.81	0.13	0.11	0.47	0.81	0.10	0.11
(E) Dressing, lower body	0.88	0.31	0.07	0.11	0.89	0.30	0.14	0.11
(F) Toileting	0.91	0.25	0.11	0.10	0.91	0.23	0.14	0.10
Sphincter control								
(G) Bladder management	0.88	0.07	0.22	0.17	0.85	0.16	0.20	0.22
(H) Bowel management	0.81	0.11	0.21	0.29	0.84	0.15	0.16	0.25
Transfers								
(I) Bed, chair, wheelchair	0.88	0.32	0.20	0.09	0.78	0.43	0.27	0.13
(J) Toilet	0.92	0.25	0.18	0.06	0.86	0.31	0.28	0.08
(K) Tub, shower	0.90	0.24	0.22	0.08	0.86	0.32	0.25	0.10
Locomotion								
(L) Walk/wheelchair	0.45	0.48	0.22	0.52	0.46	0.43	0.21	0.56
(M) Stairs	0.69	0.15	0.09	0.49	0.76	0.12	0.07	0.40
Communication								
(N) Comprehension	0.22	0.27	0.58	0.54	0.29	0.32	0.55	0.51
(O) Expression	0.05	0.22	0.50	0.70	0.08	0.39	0.24	0.78
Social cognition								
(P) Social interaction	0.22	0.35	0.44	0.63	0.29	0.29	0.50	0.59
(Q) Problem solving	0.31	0.20	0.81	0.21	0.42	0.23	0.71	0.26
(R) Memory	0.27	0.10	0.69	0.44	0.23	0.18	0.74	0.36

**Table 2:** Exploratory factor analysis of FIM motor score at the beginning and at the end of rehabilitation (Varimax rotated loadings).

FIM items	Pretest			Posttest		
	Factor 1	Factor 2	Uniqueness	Factor 1	Factor 2	Uniqueness
Self-care						
(A) Eating	0.15	0.81	0.33	0.19	0.81	0.30
(B) Grooming	0.34	0.86	0.14	0.36	0.87	0.11
(C) Bathing	0.75	0.53	0.16	0.76	0.51	0.16
(D) Dressing, upper body	0.40	0.86	0.10	0.40	0.86	0.10
(E) Dressing, lower body	0.85	0.38	0.13	0.86	0.38	0.12
(F) Toileting	0.89	0.33	0.10	0.89	0.31	0.10
Sphincter control						
(G) Bladder management	0.90	0.15	0.17	0.86	0.23	0.21
(H) Bowel management	0.82	0.19	0.29	0.84	0.21	0.25
Transfers						
(I) Bed, chair, wheelchair	0.87	0.40	0.09	0.77	0.53	0.14
(J) Toilet	0.91	0.33	0.06	0.86	0.42	0.09
(K) Tub, shower	0.90	0.33	0.09	0.85	0.42	0.10
Locomotion						
(L) Walk/wheelchair	0.43	0.52	0.54	0.45	0.49	0.57
(M) Stairs	0.68	0.21	0.49	0.76	0.17	0.40

**Table 3:** Basic characteristics of the sample.

Estimate	Mean^a^	Standard deviation^a^	Median	Range		Interquartile range		*n*
				Min	Max	25%	75%	
Age, years	58.7	15.5	61	18	87	50	69	155
Length of stay, days	24.8	19.4	17	3	107	11	32	155
Time since SCI onset, months	20.1	49.6	3.1	0.0	330.9	1.2	13.1	155
Preassessment, days after admission	2.2	1.4	2.0	0	11	1	3	155
Postassessment, days before discharge	2.7	8.3	1.0	0	68	0	2	154
FIM scores, points								
Preintervention								
Total	97.6	25.5	107	36	126	78	120	155
Motor	64.2	23.9	74	13	91	45	85	155
Cognitive	33.4	2.8	35	18	35	33	35	155
Postintervention								
Total	102.7	24.3	114	31	126	90	123	155
Motor	69.3	22.4	80	13	91	56	88	155
Cognitive	33.5	2.7	35	18	35	33	35	155

^a^All of the estimates were distributed abnormally.

## Additional Points

(i) The study used data gathered during a long time interval of seven years. (ii) The comprehensive analysis of the FIM psychometric properties inferred probable instability of the FIM factor structure over time, a topic that has been studied only little so far. (iii) Even if the results were statistically significant, the sample of 155 patients may be too small to examine an 18-item scale and to represent all people with SCI in general. (iv) The time interval of two or three weeks between the repeated measures was probably too short to detect small changes in FIM scores or the FIM factor structure. (v) This limitation might also affect the psychometric properties of FIM as the assessors, when scoring the final situation, almost certainly still recalled the initial scores resulting in reduced test-retest reliability.

## Data Access

The study protocol and statistical analysis plan are available on request from the corresponding author.
